# Predictors of adverse short-term outcomes in late preterm infants

**DOI:** 10.1186/s12887-023-04112-z

**Published:** 2023-06-17

**Authors:** Nina Mekic, Amela Selimovic, Almira Cosickic, Majda Mehmedovic, Devleta Hadzic, Evlijana Zulic, Sehveta Mustafic, Amra Serak

**Affiliations:** 1 Pediatric Department, Health and Educational Medical Center Tuzla, Tuzla, Bosnia and Herzegovina; 2 Clinic for Children’s Diseases Tuzla, University Clinical Center Tuzla, Tuzla, Bosnia and Herzegovina; 3 Clinic for Internal Medicine, University Clinical Center Tuzla, Tuzla, Bosnia and Herzegovina; 4 Polyclinic for Laboratory Diagnostics University Clinical Center Tuzla, Tuzla, Bosnia and Herzegovina

**Keywords:** Short-term outcome, Preterm birth, Late preterm infant, Intensive care unit

## Abstract

**Background:**

Infants born between 34 weeks and 36 weeks and 6 days of gestation are defined as late preterm infants (LPIs), and they account for approximately 74% of all premature births. Preterm birth (PB) remains the leading cause of infant mortality and morbidity worldwide.

**Aim:**

To analyse short-term morbidity and mortality and identify predictors of adverse outcomes in late preterm infants.

**Patients and methods:**

In this retrospective study, we evaluated adverse short-term outcomes of LPIs admitted to the Intensive Care Unit (ICU), Clinic for Children’s Diseases, University Clinical Center Tuzla, between 01.01.2020 and 31.12.2022. The analysed data included sex, gestational age, parity, birth weight, Apgar score (i.e., assessment of vitality at birth in the first and fifth minutes after birth), and length of hospitalization in NICU, as well as short-term outcome data. Maternal risk factors we observed were: age of mother, parity, maternal morbidity during pregnancy, complications and treatment during pregnancy. LPIs with major anatomic malformations were excluded from the study. Logistic regression analysis was used to identify risk factors for neonatal morbidity among LPIs.

**Results:**

We analysed data from 154 late preterm newborns, most of whom were male (60%), delivered by caesarean Sect. (68.2%) and from nulliparous mothers (63.6%). Respiratory complications were the most common outcome among all subgroups, followed by CNS morbidity, infections and jaundice requiring phototherapy. The rate of almost all of the complications in the late-preterm group decreased as gestational age increased from 34 to 36 weeks. Birth weight (OR: 1,2; 95% CI: 0,9 − 2,3; p = 0,0313**)** and male sex (OR: 2,5; 95% CI: 1,1–5,4; p = 0,0204) were significantly and independently associated with an increased risk for respiratory morbidity, and gestational weeks and male sex were associated with infectious morbidity. None of the risk factors analysed herein were predictors of CNS morbidity in LPIs.

**Conclusion:**

A younger gestational age at birth is associated with a greater risk of short-term complications among LPIs, thus highlighting the need for increased knowledge about the epidemiology of these late preterm births. Understanding the risks of late preterm birth is critical to optimizing clinical decision-making, enhancing the cost-effectiveness of endeavours to delay delivery during the late preterm period, and reducing neonatal morbidity.

## Background

Preterm birth (PTB) is defined as birth before 37 gestational weeks [1, 2]. Infants born between 34 weeks and 36 weeks and 6 days of gestation are defined as late preterm infants (LPIs), and they account for approximately 74% of all premature births and 8% of total births [3]. PTB remains the leading cause of infant mortality and morbidity. Recently, increased attention has been devoted to better understanding the reasons for the high rate of late preterm birth, its causes, its short-term sequelae, and opportunities for prevention.

Research has revealed that LPIs have a slightly but significantly higher risk of adverse outcomes than those born at term.

The duration of care for LPIs is significantly greater, since they account for a large number of neonatal deaths [4].

The physiological and metabolic immaturity of LPIs makes their compensatory responses to the extrauterine environment limited compared to those born at term and predisposes these neonates to short- and long-term complications [3, 5–10].

Common perinatal outcomes associated with late prematurity include respiratory symptoms, such as respiratory distress syndrome, transient tachypnoea of the newborn (TTN),

and neonatal pneumothorax (NP) [11–14]. Respiratory distress syndrome (RDS) is a clinical diagnosis based on signs and symptoms of increased work of breathing, tachypnoea, grunting, retractions and typical X-ray findings. RDS remains one of the most common respiratory disorders affecting LPIs [1]. The higher risk of TTN among LPI is due to the immaturity of the lung epithelium in combination with the immaturity of the epithelial Na^+^ channel (ENaC) transition as well as lower surfactant production [15, 16]. All neonates are at risk of NP; this risk is even higher for premature infants, especially those requiring mechanical ventilation [17]. Persistent pulmonary hypertension of the newborn (PPHN) is characterized by high pulmonary vascular resistance and persistent hypoxemia after birth [18]. The treatment of RDS in LPIs often requires the use of continuous positive airway pressure (CPAP) or mechanical ventilatory support [4, 19, 20].

Sepsis accounts for up to one-third of neonatal deaths worldwide each year. The World Health Organization acknowledges neonatal sepsis as a major global health concern and that the highest burden occurs in low- and middle-income countries [21]. Due to the immaturity of the immune system, LPIs are more likely to be diagnosed with culture-proven sepsis and have a higher risk of sepsis-related mortality [5, 22, 23]. LPIs demonstrate specific infection rates, pathogen distribution, and mortality associated with sepsis [24].

LPIs are more susceptible to short- and long-term neurological morbidity. The prevalences or neonatal convulsions and electrographic-only seizures are higher among preterm newborns [25–27]. Hypoxic-ischaemic encephalopathy and intracranial haemorrhage mostly account for the aetiology of preterm infants [25].

Neonatal hypoglycaemia is a common metabolic disorder that can lead to adverse effects, but it can be addressed with early diagnosis and treatment [28]. The physiologic postnatal decrease in blood glucose levels is much greater in preterm infants than in term infants due to various factors, including inadequate glycogen stores, muscle protein, and body fat needed to sustain the substrates required to meet energy needs, as well as the immaturity of enzymes involved in glucose release. Inadequate compensatory mechanisms contribute to a higher risk of developing hypoglycaemia in preterm infants than in term infants [1].

LPIs have a lower nadir hematocrit compared to term neonates and nadir hematocrit is inversely proportional to GA. Normocytic, normochromic anemia is well tolerated in some LPIs while others require blood transfusion [29].

One of the most common physiological-metabolic events in neonates is hyperbilirubinemia. Hyperbilirubinemia is caused by an increased bilirubin load secondary to a short erythrocyte lifespan, immature conjugation and excretion and increased enterohepatic circulation.

More than 80% of newborns will have some degree of hyperbilirubinemia. Acute bilirubin encephalopathy and kernicterus should be prevented by monitoring all newborns, especially LPIs [30]. A lower gestational age is a risk factor for developing significant hyperbilirubinemia (i.e., the risk of hyperbilirubinemia increases with each additional week less than 40 weeks) [31]. Serious complications of hyperbilirubinemia occur far more frequently in low- and middle‐income countries. This is due to the increased prevalence of sepsis, less accessible and developed prenatal or postnatal care, as well as a lack of resources to treat neonates with severe hyperbilirubinemia [32]. Furthermore, hyperbilirubinemia is the most common reason for readmissions in preterm infants following discharge.

The health risks for both the mother and the infant are related to advanced maternal age pregnancy disorders, chronic diseases, the need for the use of assisted reproduction technology (ART), multiple births and caesarean sections, all of which may contribute to the global increase in premature births [33].

Readmission during the neonatal period and prolonged neonatal intensive care unit (NICU) stay is a substantial societal burden and a major public health problem associated with late prematurity [2, 5, 34]. Differences in practice during birth hospitalization may affect outcomes and readmission after discharge [35].

The incidence of late preterm births and associated perinatal outcomes of those treated in the intensive care unit have not been well studied in developing countries such as Bosnia and Herzegovina. There may be differences in the number of late preterm births, as well as in their early neonatal morbidity and mortality in developed and developing regions.

Thus, the aim of the current study was to estimate the effect of gestational age on short-term neonatal morbidity and mortality in LPIs treated in the intensive care unit and to identify predictors of adverse neonatal outcomes.

## Patients and methods

A retrospective study of all LPIs (34 + 0/7 to 36 + 6/7 weeks of gestation) admitted to the Intensive Care Unit (ICU), Clinic for Children’s Diseases, University Clinical Center Tuzla (UKC Tuzla) during the three-year period from 1.1.2020 to 31.12.2022 was conducted.

The study protocol was approved by the Ethics Committee of UKC Tuzla.

LPIs treated in the ICU were identified by searching our Clinical computerized records database (BIS). LPIs with major anatomic malformations were excluded from the study. It is important to note that within the UKC Clinic for Gynaecology and Obstetrics, there is a department for newborns where LPIs that do not require an intensive level of care are treated. The strength of the study is that all infants were delivered and cared for at the same academic institution and it provides insight into the maternal risk factors. Another strength is that the data for all mothers and infants were pooled from 1 database. The limitation of our study is that it was retrospective.

The analysed data included sex, gestational age, parity, birth weight, Apgar score (i.e., assessment of vitality at birth in the first and fifth minutes after birth), and length of hospitalization in the ICU. Maternal risk factors we observed were: age of mother, parity, maternal morbidity during pregnancy, complications and treatment during pregnancy.

Short-term outcome data were divided into four categories: respiratory morbidity, neurological morbidity, infectious morbidity and additional outcomes.

Respiratory morbidity was defined as any of the following: the presence of respiratory distress syndrome (RDS), transient tachypnoea of the newborn (TTN), persistent pulmonary hypertension of neonate PPHN, NP or the need for ventilatory support such as mechanical ventilation and continuous positive airway pressure (CPAP). Culture-proven sepsis, pneumonia and meningitis were classified as infectious morbidities. Neonatal convulsions and intraventricular haemorrhage (IVH) were classified as central nervous system (CNS) morbidities. Additional morbidities included jaundice requiring phototherapy (indication for phototherapy based on guidelines from AAP 2004 [36]), hypoglycaemia (blood glucose level of less than < 2,2 mmol/L (40 mg/dL) in capillary or venous blood sample [9, 37]) and anaemia requiring blood transfusion.

Infant outcomes were based on clinical diagnoses made by a paediatric physician in accordance with diagnostic protocols, with ranges defined by the Department of Paediatrics.

In addition to clinical assessment, radiological diagnostic tools were used for respiratory pathology, as well as for neurological pathology. In the assessment of infection and entities that are classified as additional morbidities, laboratory and microbiological findings were used. The criteria for the diagnosis of neonatal sepsis required isolation of the microorganism from a blood culture and at least one clinical sign or symptom [38].

Statistical data analysis was conducted using the biomedical software application “MedCalc for Windows, Version 15.11.4” (MedCalc Software, Ostend, Belgium). Distribution of variables determined by the Kolmogorov‒Smirnov test. The variables with distorted distribution are shown with a median as a measure of the central value. Student’s *t* test and one-way analysis of variance were used to compare continuous variables between the groups, and *X*^2^ and Mann‒Whitney *U* tests were used for categorical variables. Multivariable logistic regression analysis was used to identify risk factors for adverse outcomes among late preterm infants. For the purpose of subgroup analysis in the late preterm group, the gestational week group was defined as the number of completed weeks of gestation. Thus, an infant born at a gestational age of 35 weeks and 6 days was included in the 35-week group. Differences were considered significant when P < 0.05.

## Results

During the study period, there were 8544 live births at the University Hospital Tuzla at the Clinic for Gynaecology and Obstetrics, of which 842 (9.85%) were preterm infants, including 504 (5.89%) LPIs. A total of 674 infants were admitted to the NICU of the Clinic for Children’s Diseases, University Clinical Center Tuzla, 430 (63,8%) preterm and 244 (36.2%) term infants. Out of 430 preterm infants treated in the ICU, one-third were late preterm (154; 35.2%), and out of 504 LPIs born in the studied period, 154 (30.5%) needed intensive care and treatment. LPIs were more likely to be males, delivered by caesarean section (CS) and from nulliparous mothers. Most LPIs were born at 34 gestational weeks (72; p = 0.0013) (Table 1).

**Table 1:** Demographic and obstetric characteristics of LPIs

	Late Preterm (n = 154)				
	Overall n (%)	**34 wk (n = 72)**	**35 wk (n = 43)**	**36 wk (n = 39)**	P value
Multiparity	55 (35.7)	24 (33.3)	16 (37.2)	15 (38.5)	0.0475
Nulliparity	99 (64.3)	48 (66.7)	27 (62.8)	24 (61.5)	0.0475
Gestational age at delivery (wk)	154	72 (46.8)	43 (27.9)	39 (25.3)	**0.0013**
Cesarean delivery	105 (68.2)	51 (68.0)	26 (60.5)	28 (72)	0.4386
Birth weight (g)	2518±585	2236±414	2570±464	2985±660	
Birth lenght (cm)	50 (39–59)	48 (39–54)	51 (46–56)	53 (48–59)	
Head circumference (cm)	33 (29–37)	32 (29–34)	32 (30–34)	34 (31–37)	
*Apgarscore*					
1st minute	8 (1–9)	8 (3–9)	8 (5–9)	8 (1–9)	0.090
5th minute	8 (8–9)	8 (7–9)	8 (8–9)	8 (7–9)	**0.020**
Male/Female	93(60)/61(40)	41(56,9)/31 (43.1)	27 (62.8)/16 (37.2)	25 (64.1)/14 (35.9)	0.7462

Data are mean±standard deviation, number, or median (interquartile range)

* Refers to comparison of late preterm subgroups: 34 compared with 35 compared with 36 weeks

The analysis of maternal factors and late preterm birth is shown in Table 2. Maternal obstetrical factors included hypertensive disorder of pregnancy HDP (25.9%), anaemia (32.5%), hypothyroidism (5.2%), diabetes (9.7%) and infection (colpitis and vulvovaginitis, 14.9%). HDP includes chronic hypertension, pregnancy-induced hypertension, and preeclampsia. Diabetes as maternal factor included gestational and established diabetes. Other data that we presented in our study are: use of steroids (65.6%), ART (16.9%), preterm premature rupture of the membranes (PPROM) (41.6%), placental ablation (3.2%), placenta preavia (1.9%) and other obstetric factors (54.5%). Under other factors we included: polyhydramnios or oligohydramnios, fetal distress, previous cesarean section, breech presentation and maternal diseases not previously specified. Approximately 79% of mothers who had a late preterm birth had at least one of the above maternal diseases. Urgent caesarean sections was most common birth way (64.9%), followed by vaginal delivery with induction – stimulation of labour (29.9%), elective caesarean Sect. (3.2) and spontaneous vaginal delivery (1.9%).

**Table 2:** Association of maternal factors and late preterm birth

Variable	Maternal / prenatal / labour characteristics		
		Frequency	Percentage
Age of mother	< 20 20–35 > 35	6 138 10	3.9 89.6 6.5
Multiparous	2–5 > 5	55 3	35.7 1.9
Maternal morbidity during pregnancy, other complications and treatment during pregnancy	None HDP Use of steroids Anaemia Hypothyroidism Diabetes Infection ART PPROM Placental ablation Placenta preavia Other	31 40 101 50 8 15 23 26 64 5 3 84	20.1 25.9 65.6 32.5 5.2 9.7 14.9 16.9 41.6 3.2 1.9 54.5
Birth way of the newborn	Spontaneous vaginal delivery Vaginal delivery with induction Elective caesarean sections Urgent caesarean sections	3 46 5 100	1.9 29.9 3.2 64.9

Respiratory complications were the most common outcome in all subgroups, followed by CNS morbidity, infections and jaundice requiring phototherapy. The rate of almost all of the complications in the late-preterm group decreased as gestational age increased from 34 to 36 weeks. Nine neonatal deaths occurred (6%), including the same number of deaths at 34 and 36 weeks (4 infants each) (Table 3).

**Table 3:** Neonatal outcomes in late preterm infants

	Late Preterm (n = 154)				
	Overall n (%)	34 wk (n = 72)	35 wk (n = 43)	36 wk (n = 39)	P value
Hospitalization (d)	6 (4–8)	6±3	9±6	6 (4–12)	
Hospitalization≥10 d	27 (17.5)	11(15.3)	7 (16.3)	9 (23.1)	
Neonatal death**	9 (6.0)	4 (6.0)	1 (2.0)	4 (10.3)	0.3076
***Respiratory morbidity***					
RDS	115 (74.7)	51(70.8)	33 (78.6)	31 (79.5)	0.5664
TTN	4 (2.6)	2 (2.8)	0	2 (5.1)	0.3424
Mechanical ventilation	13 (8.4)	4(5.6)	2 (4.7)	7 (17.9)	**0.0456**
CPAP	35 (22.7)	17 (23.6)	10 (23.8)	8 (20.5)	**< 0.001**
Persistent pulmonary hypertension	28 (18.2)	14 (19.4)	4 (9.3)	10 (25.6)	0.1485
Pneumothorax	13 (8.4)	6 (8.3)	1 (2.3)	6 (15.4)	0.1046
***Infectious morbidity***					
Sepsis	35 (22.7)	12 (16.7)	9 (20.9)	14 (35.9)	0.0660
Pneumonia	30 (19.4)	13 (18.1)	6 (13.9)	11 (28.2)	0.2438
Meningitis	4 (2.6)	3 (4.2)	0	1 (2.6)	0.3945
***CNS morbidity***					
Convulsions	6 (3.9)	2 (2.8)	3 (7.0)	1 (2.6)	0.4688
IVH grade 1–2	73 (47.4)	35 (48.6)	20 (46.5)	18 (46.2)	0.9670
IVH grade 3–4	4 (2.6)	4 (5.6)	0	0	0.0965
***Additional morbidities***					
Jaundice requiring phototherapy	69 (44.8)	36 (50)	18 (41.9)	15 (38.5)	0.4559
Hypoglycemia	41 (26.6)	22 (30.6)	7 (16.3)	12 (30.8)	0.1951
Anemia requiring blood transfusion	17 (11.0)	11 (15.3)	4 (9.3)	2 (5.1)	0.2421

RDS, respiratory distress syndrome; TTN, transient tachypnea of the newborn; CPAP, continuous positive airway pressure; CNS, central nervous system; IVH, intraventricular hemorrhage. Data are mean±standard deviation or median (interquartile range)

We aimed to identify the risk factors for neonatal morbidity among LPIs. Using multivariable logistic regression analysis, only nulliparity was significantly and independently associated with an increased risk of adverse neonatal outcomes. Additionally, birth weight and male sex were significantly and independently associated with an increased risk of respiratory morbidity, and gestational weeks and male sex were associated with the risk of infectious morbidity. None of the risk factors analysed herein were predictors of CNS morbidity among LPIs (Table 4).

**Table 4:** Factors predicting adverse neonatal outcomes for late preterm infants

	Adverse neonatal outcome	Respiratory morbidity	CNS morbidity	Infectious morbidity
Gestational wk	1.324 (0.831–2.110) p = 0.2374	1.013 (0.590–1.737) p = 0.9625	1.038 (0.590–1.737) p = 0.8830	2.030 (1.223–3.370) p = 0.0061*
Male sex	0.8814 (0.454–1.707) p = 0.7082	2,501 (1.152–5.429) p = 0.0204*	1.291 (0.628–2.655) p = 0.4863	3.427 (1.651–7.112) p = 0.0009*
Birthweight	0.9997 (0.999–1.004) p = 0.4203	1.235 (0.998-2,347) p = 0.0313*	1.000 (0.999-1.000) p = 0.9180	0.999 (0.998- 1.000) p = 0.0791
Nulliparity	0,4585 (0.236–0.889) p = 0.0211*	0,920 (0.427–1.978) p = 0.8313	1.034 (0.503–2.123) p = 0.9274	1.020 (0.502–2.070) p = 0.9557
Cesarean delivery	0.570 (0.2813–1.1589) p = 0.3009	1.663 (0.753–3.673) p = 0.2078	0.873 (0.401-1.900) p = 0.7339	1.681 (0.783–3.606) p = 0.1820

Data are the results of multivariable logistic regression analysis and are expressed as odds ratios (95% confidence intervals). *Significant results

**Fig. 1:**
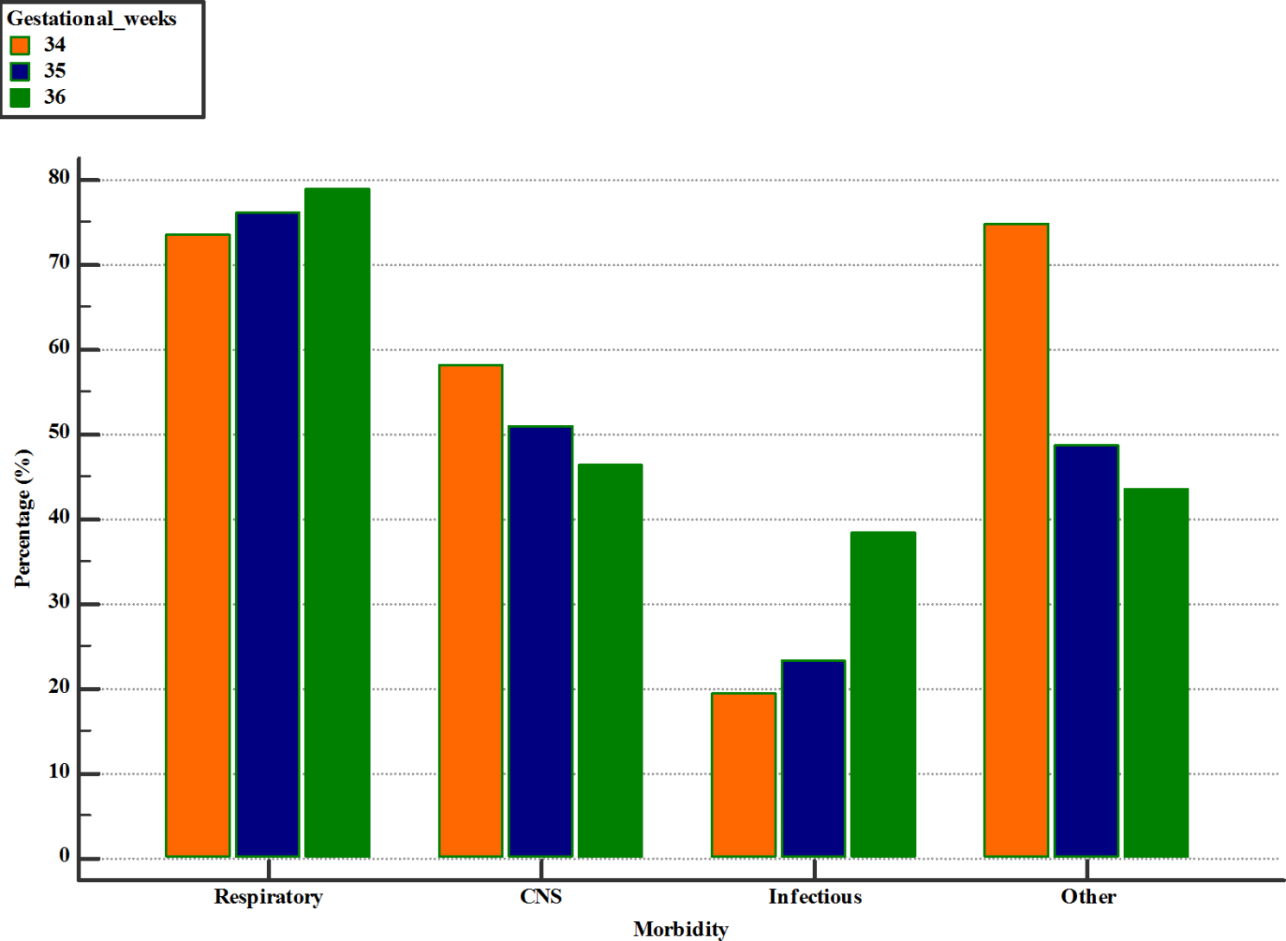
Neonatal outcomes in late preterm infants

The regression model shows that the frequency of respiratory and infectious diseases increased with gestational age, while the frequency of CNS and other diseases decreased with increasing GA (Fig. 1).

## Discussion

Globally, the increase in the incidence of premature birth is almost entirely due to infants born late preterm [39].

The increase in late-preterm births may be attributed to the increasing use of ART. ART is associated with adverse maternal and newborn outcomes [40, 41]. The progress in obstetric practices and early recognition of foetal endangerment, more frequently result in the completion of pregnancies with infants born late preterm [42].

Of the 8544 infants born in the three-year period, there were 842 (9.85%) preterm infants, including 504 (5.89%) LPIs. Of these, 154 LPIs required intensive care and therapy in the ICU, i.e., 1.8% of all infants, 18.2% of preterm infants or 30.5% of all LPIs born in the studied period.

It is important to note that within the UKC Clinic for Gynecology and Obstetrics, there is a department for newborns where LPIs who do not require an intensive level of care are treated. In the USA, the preterm delivery rate is 12–13%; in Europe and other developed countries, the reported rates generally range from 5 to 9% [33, 43]. Increases in the singleton preterm birth rate are almost entirely due to infants born late preterm [39].

The results of our study showed that preterm infants were predominantly treated in the ICU, including 430 (63.8%) preterm and 244 (36.2%) term infants. Out of the 430 preterm infants treated in the ICU, one-third were late preterm (154; 35.2%), and the majority of them were male, similar to other studies [19, 35, 44].

Of the 154 preterm infants included in our study, 104 (68.18%) were born by CS. A higher CS rate was also reported by Tsai et al. and Ma et al. [9, 37]. On the other hand, Champion et al. [45], Aliaga et al. [35] and Liqun Lu et al. found that vaginal delivery, especially with the induction of labour, was the main mode of delivery of late preterm infants (59.6%) [46].

When making decisions regarding obstetric interventions, such as induced labour, vaginal delivery or CS, the risks of continuing the pregnancy in a suboptimal uterine environment must be compared with the risks of early delivery [47].

Neonatal morbidity is significantly higher in combination with maternal medical conditions, with the independent effect of late preterm birth on early neonatal morbidity being nearly seven times greater than the independent effect of maternal risk factors [48].

Rate od LPIs was highest among women of < 20 and > 35 years of age and lowest among women of 20–34 years of age. Due to an increased prevalence of diabetes and hypertension, as well as higher use of ART, older women may be in increased risk of having preterm birth [41]. Increased preterm risk among teens may be due to biologic immaturity, socioeconomic status, and different life habits [22, 49]. 3.9% and 6.5% of mothers who delivered late preterm infants were aged < 20 years and ≥ 35 years, respectively, compared to the study in China where 4.5% and 8.4%. There is also a difference in the method of delivery. In their study vaginal was dominant, while in ours it was an emergency caesarean section. The mentioned difference may be due to the inclusion of all late preterm infants in their study, not only those treated in the ICU [46]. Differences in the incidence of hypertension, preeclampsia and PROM can be interpreted in the same way.

It is known that birth weight in relation to gestational age affects the morbidity and outcomes of late-preterm infants [50]. The average birth weight in our study was 2518 g and was between the 3rd and 97th percentiles of birth weight for gestational age.

Preterm birth is not a single entity but a common final outcome of underlying maternal and foetal factors. Parity (i.e., the number of offspring a female has delivered) was also found to be associated with adverse birth outcomes [51, 52]. For nulliparous mothers, late-preterm birth was significantly and independently associated with an increased risk for adverse neonatal outcomes, similar to a recent study with 837,226 singleton births conducted in the Netherlands. The mentioned study showed that the risks of PTB were significantly higher in nulliparous mothers than in mothers who had given birth at least once (RR: 1.95, 95% CI: 1.89–2.00 for PTB) [53]. In the results presented by Torres-Munoz, 41.04% of the group of cases with LPIs were primiparous women, and 54.72% were multiparous women (2–5 births), compared to the control group in which primiparous women were represented in a smaller percentage, i.e., 37.26% [54]. However, a systematic review consisting of 41 studies did not reach the same conclusion [55]. Additionally, significant heterogeneity was found among the included studies, indicating the need for more studies.

A study by De Luca et al. [27] revealed a strong age-related trend in respiratory morbidity independent of delivery mode and a > 10-fold increase in respiratory morbidity in infants born at 34 weeks GA compared with those born at term. A retrospective study in Canada by Kitsommart et al. [28] revealed significantly worse respiratory outcomes (including the prevalence of NP and the rates of positive pressure therapy, and mechanical ventilation assistance; all P < 0.001) in 1481 infants born at 34 to 36 weeks GA compared with 9332 infants born at ≥ 37 weeks GA.

The risk of adverse short-term outcomes changes with each week of gestation [19, 45, 56, 57]. Our results were fairly consistent, and the rate of almost all complications in the late-preterm group decreased as gestational age increased from 34 to 36 weeks.

Adverse short-term respiratory outcomes were the most common complications at all gestational ages. Infants born at 34 weeks had 40-fold increased odds of developing RDS versus infants born at 39 weeks [58]. In our study, the rates of RDS were 74,7%, 70,8% and 7.,6% for infants born at 34 weeks GA, 35 weeks GA and 36 weeks GA, which was similar to the rates of 70.3%, 79.5% and 70.65% in the study by McIntire et al. [11] but significantly higher than those of 22.3%, 31.5% and 32.5% in the study by Jones et al. [59]. Respiratory support was needed for up to 58,1% of infants admitted to the NICU according to Aliaga et al. [35]. The need for mechanical ventilation followed the rate of RDS in our study and was the highest in the 36th gestational week. We had more LPIs on MV at 36 weeks GA because 3 LPIs had sepsis, 1 had severe asphyxia, and 2 had a severe form of RDS with pneumothorax.

The studies by Tutdibi et al. and Derbent et al. indicate that TTN is strongly related to elective CS delivery and low GA [15, 16]. The rates of TTN and NP in our study were similar at 34 and 36 weeks GA and were present exclusively in neonates born by CS. In a study performed in 2017–2019 in the same clinic as our study, a lower gestational week and CS delivery were shown to be risk factors for NP in neonates on mechanical ventilation [60]. For PPHT, the vast majority of infants with PPHN were born at term or near term, although approximately 2% of cases were born prematurely [61]. Our study supports the claim that the highest incidence of PPH occurs at the highest GA, i.e., 25.6%.

In LPIs, treatment for suspected systemic infection is far more common than for term infants, and the diagnosis is made on the basis of clinical symptoms, laboratory findings and/or positive blood cultures [44]. Sepsis was diagnosed in 22.7% of the late-preterm infants in our study, and the literature mentions infection frequency percentages of 4,9–20,6%[11, 62]. The pattern of pathogen distribution differs from region to region as well as between developed and less developed countries due to patient demographic characteristics, the colonization of the microflora of the hospital environment and antibiotic use policies.

[38], which may explain the higher rate of sepsis in our study. Pneumonia was present in 18.8% of the LPIs, while according to Loftin et al., the frequency was 1.2% in newborns at 34 weeks GA; an overall rate of 0.7% was described in the study by Melamed et al. [7, 40], in which 2.6% of the LPIs had meningitis.

Regarding the short-term CNS morbidity outcomes, neonatal convulsions accounted for 3.9% of the LPIs in our study. Although the incidence of severe intraventricular haemorrhage is low in LPIs when compared to other preterm infants, LPIs are still at higher risk when compared to term neonates. The large variation in the reported rates of IVH is due to the lack of standard guidelines for screening neuroimaging in LPIs, so we can elucidate the reason for the high number of LPIs with IVH grade 1 and 2 in our study. McIntire et al. reported rates of IVH grade 1 and 2 in LPIs of 0.5% at 34 weeks GA, 0.2% at 35 weeks GA and 0.06% at 36 weeks GA, but when we analyse only at the data on LPIs admitted to the NICU, the percentage increases to 10.9% at 34 weeks GA. Teune et al. reviewed 22 studies and found that intracranial haemorrhage occurred more frequently in LPIs. The rate of either IVH grade 3 or 4 was extremely low in LPIs [11]; however, it remained higher than that in term neonates (0.01% vs. 0.004%) [63]. Our rate of IVH grade 3 or 4 was higher, at 2.6%, and occurred only in the 34th week of gestation. This percentage was expectedly higher, considering that Teune et al. reviewed the data of all LPIs, not only those admitted to the NICU.

The most common additional short-term outcome in our study was hyperbilirubinemia, defined as jaundice that required phototherapy, as in multiple studies in which it occurred during initial hospitalization [11, 64, 65]. Very high rates of jaundice were reported by Wang et al., and more than half of the LPIs in a large-based practice report developed hyperbilirubinemia requiring phototherapy [12, 66]. Aliaga et al. reported that 33.2% of late-preterm newborns in the study group received phototherapy. More late-preterm newborns admitted to the NICU (58.3%) received phototherapy than those admitted to the newborn nursery (19.9%). Phototherapy was more common in infants born at 34 weeks GA than at 35 and 36 weeks GA (63.5 vs. 34.7 vs. 21.2%). LPIs who were treated for hyperbilirubinemia in the same way as term infants developed kernicterus and had more sequelae from hazardous hyperbilirubinemia [67]. Existing guidelines are helpful for treating hyperbilirubinemia in infants who are born at 35 weeks GA or later.

As a result of metabolic immaturity, hypoglycaemia is common among LPIs, with an overall incidence of up to 50% [7, 37, 64, 66, 67]. Our study, with a total of 26% of infants with hypoglycaemia, confirms the metabolic immaturity of preterm infants and is consistent with the results of the aforementioned studies. A meta-analysis further confirmed the increased risk of hypoglycaemia in LPIs compared with term infants [63].

In our study, 11,0% of LPIs had anaemia requiring blood transfusion, which was expected considering the morbidities of preterm infants requiring admission to the NICU. The rate of anaemia decreased as gestational age increased from 34 to 36 gestational weeks.

LPIs not requiring intensive treatment are treated at the Neonatal Department of the Clinic for Gynecology and Obstetrics. Neonates born at 35 weeks GA and 36 weeks GA who required intensive care and therapy were included in this study, which provides an explanation for the greater number of respiratory and infectious diseases in infants with older gestational age.

## Conclusion

Identifying the predictors of adverse short-term outcomes in late preterm infants is of crucial importance for informing and evaluating clinical practices and guidelines aimed at reducing infant morbidity and mortality. The differences in the number of late-preterm births, as well as in the early neonatal morbidity and mortality rates in developed and developing regions, should stimulate more research to analyse risk factors for latepreterm births in underdeveloped regions.

## Data Availability

The datasets generated and analyzed during the current study are available from the corresponding author on reasonable request.

## Abbreviations

LPIs: Late preterm infants
PTB: Preterm birth
ICU: Intensive Care Unit
UKC Tuzla: University Clinical Center Tuzla
BIS: Clinical computerized records database (BIS)
NICU: Neonatal Intensive Care Unit
RDS: Respiratory distress syndrome
TTN: Transient tachypnea of the newborn
CPAP: Continuous positive airway pressure
IVH: Intraventricular hemorrhage
CNS: Central nervous system
HDP: Hypertensive disorder of pregnancy
ART: Assisted reproduction technology
